# Application of Sensor Technology in Wheelchair Sports for Real-Time Data Collection during Training and Competition and for Assessment of Performance Parameters: A Systematic Review and Future Directions

**DOI:** 10.3390/s24196343

**Published:** 2024-09-30

**Authors:** Yehuda Weizman, Lena Bäumker, Franz Konstantin Fuss

**Affiliations:** 1 Chair of Biomechanics, Faculty of Engineering Science, University of Bayreuth, D-95440 Bayreuth, Germany; yehuda.weizman@uni-bayreuth.de (Y.W.); lena.baeumker@uni-bayreuth.de (L.B.); 2 Division of Biomechatronics, Fraunhofer Institute for Manufacturing Engineering and Automation IPA, D-95447 Bayreuth, Germany

**Keywords:** wheelchair sports, sensor, inertial measurement unit (IMU), accelerometer, gyroscope, performance parameters, biomechanical parameters, match time, competition

## Abstract

This review reports on the use of sensors in wheelchair sports to monitor and analyze performance during match and training time. With rapid advancements in electronics and related technologies, understanding performance metrics in wheelchair sports is essential. We reviewed nine studies using various sensor types, including electric motors, inertial measurement units, miniaturized data loggers with magnetic reed switches, and smartphones with inbuilt accelerometers and gyroscopes, operating at frequencies from 8 Hz to 1200 Hz. These studies measured parameters such as angular and translational velocities, distance, number of starts/pushes, and other performance indicators in sports such as basketball, rugby, tennis, and racing. Despite differences in sport types and methodologies, most studies found sensor-derived data effective for assessment of performance. Future developments and research in this field should focus on multi-sensor systems that could provide real-time match analysis and deeper insights into performance metrics. Overall, sensor technologies show significant potential for improving wheelchair sport performance diagnostics, contributing to better athlete training and future wheelchair design, and enhancing competitive outcomes. This review emphasizes the need for continued innovation and standardization in applying sensor technologies in wheelchair sports.

## 1. Introduction

The popularity of wheelchair sports has significantly increased in recent years, with sports such as wheelchair basketball, racing, rugby, and tennis gaining traction and audience interest [1]. Recent technological advances, specifically in motion and tracking sensors such as inertial measurement units (IMUs), have greatly improved sport performance analysis. These devices, which incorporate triaxial microsensors such as gyroscopes, accelerometers, and magnetometers, provide extensive opportunities for biomechanical studies in sports [2]. Recent advancements in material and manufacturing technologies have also significantly impacted wheelchair optimization and customization to enhance sport performance. Manufacturers now design these products using anthropometric data from individual athlete users, ensuring a precise and personalized fit [3,4].

While numerous studies [5,6,7,8,9,10,11,12,13,14] in wheelchair sports have investigated controlled-environment protocols to measure performance parameters using various methods, such as ergometer and treadmill [5,14], selected and staged training activities [10,11,13], and force plate technologies [5], the literature on measuring these parameters during real standard training and competition is relatively limited. Assessing performance during simulated and actual match play is crucial for several reasons. Real and simulated match conditions provide an authentic setting where athletes face the challenges and pressures of competition, allowing for a more accurate assessment of performance under dynamic and unpredictable circumstances. This environment enables researchers and coaches to analyze and reveal pragmatic and critical metrics related to velocity, agility, and tactical effectiveness, essential for understanding athlete performance in real-time training and match settings [15]. Although there are technical challenges in implementing and analyzing real-time training and match data, these provide researchers and coaches with a wider array of kinematic performance metrics related to athletes’ activities during games. Such metrics include the number of passes, blocks, turnovers, steals, and scores, offering more comprehensive insights than assessments conducted in controlled environments. Therefore, focusing on training and match time performance analysis with sensor technology not only enhances the validity of the research findings but also holds the potential to directly contribute to improving strategy development and overall competitiveness in adaptive sports.

Other studies conducted during real training and match time have utilized the integration of inertial measurement units (IMUs), electric motors/generators, and miniaturized data loggers (MDLs) with magnetic reed switches to provide detailed kinematic data for assessing various aspects of wheelchair sport performance [16,17,18,19,20,21,22,23,24]. Van der Slikke et al. [17] demonstrated the utility of three IMUs in capturing mobility performance in wheelchair basketball, highlighting their ability to measure and display six key kinematic outcomes that accurately describe wheelchair mobility performance in match play. Another study by Chua et al. [22] on activity identification and classification in wheelchair rugby used an accelerometer attached to the frame of a rugby wheelchair and fractal dimension analysis methods to produce a two-dimensional mapping that identifies five different types of activities during a match: no activities, low-level activities, high-speed coasting, high-speed pushing, and collisions.

Monitoring the activities and performance metrics of wheelchairs and athletes during real-time training and match competitions provides deep insights into game dynamics and athlete exertions [15]. This systematic review aims to provide an updated framework of the current research on the use of sensor technology for performance analysis in wheelchair sports during training and match time. Additionally, it explores future advancements and applications, offering insights into how emerging technologies can impact performance analysis in this field.

## 2. Methods

### 2.1. Search Strategy

A literature review was performed to identify the most relevant quantitative and qualitative studies following the Preferred Reporting Items for Systematic Reviews and Meta-Analyses (PRISMA) guidelines [25]. The electronic databases PubMed, Google Scholar, Elsevier (ScienceDirect), MDPI, Springer Nature, Wiley, ISBS Conference Proceedings Archive, and Taylor and Francis were queried for articles published until 31 May 2024. The search terms employed included (wheelchair) AND (IMU OR inertial measurement unit OR gyroscope OR acceleration).

### 2.2. Study Selection Strategy

Following the detection and removal of duplicate manuscripts, three authors (Y.W., L.B. and F.K.F) independently screened the titles, abstracts, and keywords of the records identified through the database search. If a record appeared relevant, or if its relevance was not immediately clear, the full text of the article was saved for further consideration in this review.

Articles were included if they met the following criteria:

(1) Published in English.
(2) Full original English peer-reviewed research articles.
(3) Studies focusing on the use of motion and tracking sensors to measure performance parameters in wheelchair sports during standard training and/or match time.
(4) Protocols involving experienced athlete participants.

We defined the following exclusion criteria to simplify the study selection and classification of the retrieved papers:

(1) Studies that did not include subjects with disabilities.
(2) Protocols involving staged and controlled drills for pure research purposes.
(3) Protocols involving treadmill or ergometer drills.
(4) Studies focusing on robot-assisted movement.

### 2.3. Data Extraction

Three authors (Y.W., L.B. and F.K.F) analyzed the articles, extracting the following information into three key tables: Table 1 details the study characteristics, including the aim, type of sport, participant experience, and participant characteristics. Table 2 outlines the sensor type and specifications, sensor location, assessment protocol, and key findings. Table 3 includes the sensor data type (e.g., acceleration, gyration) and calculated performance parameters.

### 2.4. Methodological Quality

This review consolidates studies that utilize sensors in wheelchair sports during training or matches. The quality of each included article was evaluated using a custom quality assessment worksheet (Table 4). The reviews by Benson et al. [26] and Campos et al. [27] served as the basis for developing a quality assessment checklist. This quality assessment comprises 13 items distributed across four subscales: reporting, external validity, internal validity (bias), and power analysis. Two authors (Y.W. and L.B.) independently evaluated the methodological quality of each study included in this systematic review.

**Table 1:** Study characteristics.

Author	Aim	Type of Sport	Participant Experience	Participant Characteristics
Fuss and Ow [16]	To develop a flexible and low-cost instrumentation system for on-track monitoring of wheelchair velocity and associated parameters during training of wheelchair racing.	Wheelchair Racing	A T53 athlete won multiple international medals, and a T52 athlete won three golds at the 2008 ASEAN Para Games.	N: 2; Gender (M/F): 1/1; Mean Age: 25 ± 6
Van der Slikke et al. [17]	To compose an easy-to-interpret display of key features representing wheelchair mobility performance. To do so, three key steps were taken: (1) Reducing many kinematic outcomes to key ones. (2) Displaying key kinematic features concisely for coaches and athletes. (3) Testing if key features distinguish between performance levels.	Wheelchair Basketball	National- and international-level athletes. Participation in premier division competitions and friendly international-level matches.	National Male (NM) N: 12; Mean Age: 27.9 ± 9.4; International Male (IM) N: 8; Mean Age: 30 ± 6; International Female (IF) N: 9; Mean Age: 28.3 ± 8.8
Rietveld et al. [18]	To determine the key IMU-based components of wheelchair mobility performance during wheelchair tennis matches.	Wheelchair Tennis	Dutch wheelchair tennis players, including eleven adult players and four talented youth players, all with ITF rankings, trained 4–15 h per week.	Adult group N: 11; Gender (M/F): 5/6; Mean Age 28.2 ± 7.1; junior group N: 4; Gender (M/F): 1/3; Mean age: 15.9 ± 1.3
Mason et al. [19]	To compare the validity and reliability of a miniaturized data logger and a radio-frequency tracking system (ITS) in quantifying mobility performance during wheelchair rugby matches.	Wheelchair Rugby	Elite male wheelchair rugby players, members of a national wheelchair rugby squad.	N: 11; Gender: M/F: 11/0; Mean age: 26 ± 6 years
Sporner et al. [20]	To quantify and compare the activity profiles of wheelchair basketball and rugby players using a wearable mobility performance tool.	Wheelchair Basketball and Wheelchair Rugby	Participants had to be 18 years of age or older and participating in wheelchair basketball or rugby. All athletes were military veterans.	Basketball: N: 20; Gender: M/F: 19/1; Mean age: 38.75 ± 10.92; Rugby N: 18; M/F: 17/1; Mean age: 41 ± 10.33
Van der Slikke et al. [21]	To evaluate the effectiveness of a wearable device for monitoring wheelchair mobility performance (WMP) across different wheelchair sports. The study seeks to improve classification methodology and increase performance insights.	Wheelchair Basketball, Wheelchair Rugby, and Wheelchair Tennis	Elite level athletes in wheelchair basketball, rugby, and tennis	Basketball N: 29; M/F: 20/9; Mean Age: Not reported; Rugby N: 32; M/F: 29/3; Mean Age: Not reported; Tennis N: 15: M/F: 4/11; Mean Age: Not reported
Chua et al. [22]	This study’s objectives are as follows: (1) Develop a two-dimensional activity identification and classification method using fractal dimension analysis (Hausdorff dimension DH and Rényi entropy S0). (2) Ensure accurate boundary lines between ranked activities through sensitivity analysis. (3) Optimize the mapping method by determining the best window width size and amplitude multiplier for DH and S0. (4) Apply the optimized method to wheelchair rugby match data to support coaches.	Wheelchair Rugby	Experienced athletes from three different levels: high-pointer, mid-pointer, and low-pointer)	N: 5; Gender: M/F: 5/0; Mean Age: Not reported
Fuss [23]	This paper aims to provide an overview of simple, yet sufficiently accurate, wheelchair speed measurements, speed data processing and analysis, performance parameters of athletes and wheelchairs, and decision-making and training intervention.	Wheelchair Racing	Paralympic Games-level athletes	N: 4; Gender: M/F: 3/1; Mean Age: Not reported
Coutts [24]	To collect data on wheelchair basketball players during coast-down and maximal-effort sprint trials and game conditions.	Wheelchair basketball	National-level athletes	N: 4 Gender M/F: 2/2 Mean age: Not reported

**Table 2:** Study methods and key findings.

Author [Ref’]	Sensor Type and Specifications	Location	Assessment Protocol	Main Findings
Fuss and Ow [16]	Electric motor: ACC337; sampling rate: 1200 Hz.	The frame of the wheelchair, driven by one of the rear wheels	Each athlete performed two 100 m sprints.	The study found that Athlete 1 (class T53) reached a maximum velocity of 8 m/s, twice that of Athlete 2 (class T52). Athlete 1 also produced 6 times the average peak push power and 2.7 times more energy over 100 m compared to Athlete 2. Additionally, Athlete 1’s push frequency decreased from 2.6 Hz to 1.3 Hz, while Athlete 2 maintained a constant push frequency of 2 Hz.
Van der slikke et al. [17]	3 IMUs: X-IO technologies; sampling frequency: not specified	Rear wheels axis; rear frame bar	The kinematics of wheelchair basketball athletes were measured during 11 premier division competitions and friendly international matches. Twenty-nine athletes were involved in the study, including twelve male first-division athletes (National NLD), nine female internationals (NLD and GBR), and eight male internationals (NLD, ISR, and AUS). Athlete classification was evenly distributed across these three competition-level groups.	Six key kinematic outcomes (Table 3) were identified that effectively describe (*p* < 0.05) wheelchair mobility performance, allowing comparisons between individual athletes and across sports.
Rietveld et al. [18]	3 IMUs: X-IO technologies; sampling frequency: 100 Hz.	Hub of each wheel; the frame bar of the wheelchair	Participants completed 64 wheelchair tennis matches (11 juniors, 25 women, 28 men) on indoor hardcourts, each playing 2 to 8 matches. IMU’s data collection was taken during the 2018 and 2019 ABN-AMRO tournaments and the Dutch National Championships using IMU-instrumented wheelchairs.	Wheelchair mobility performance in wheelchair tennis can be described by six components: (1) rotations to the racket side in curves, (2) rotations to the racket side in turns, (3) linear accelerations, (4) rotations to the non-racket side in turns, (5) rotations to the non-racket side in curves, and (6) linear velocities. These key outcome variables can be monitored to assess performance.
Mason et al. [19]	Miniaturized data logger with 2 magnetic reed switches (120 deg resolution): 10 Hz; indoor tracking system (ITS) Ubisense; 8 Hz	MDL: Attached near the axle of both left and right main wheels. ITS: Worn on the participant’s body via a vest.	Data were collected from four simulated wheelchair rugby matches over 4 × 8 min quarters using MDL and ITS from 3 to 5 participants, analyzing total distance, mean and peak speeds, and time spent in five relative speed zones derived from peak speed. Additionally, time and distance in 8 speed zones (0 to 4 m/s) were analyzed.	No significant differences in mean speed or distance covered between MDL and ITS. MDL underestimates peak speeds and shows substantial random errors in high-speed zones, questioning its use for performance monitoring in training programs.
Sporner et al. [20]	Miniaturized data logger; sampling rate: not reported	Wheel spokes	Data were collected during the NVWG Annual Wheelchair Sporting Tournament for both sports. Since the number of games recorded for each participant varied according to how far they progressed in the tournament, the first two recorded games were selected for investigation.	Significant differences in mobility patterns between basketball and rugby performance activities (Table 3), with basketball showing higher average speeds and distances covered. “Previous research has not reported basketball or rugby gameplay variables such as these, making this data set unique. ”
Van der Slikke et al. [21]	Wheelchair basketball: 3 IMUs: X-IO technologies; sampling rate: not reported. Wheelchair rugby and tennis: 3 IMUs: Shimmer3; sampling rate: not reported	Rear wheels axis; rear frame bar	Wheelchair Basketball: Measured during eleven premier division competition matches and friendly international matches; Wheelchair Rugby: Measured during the Dutch national championship of 2016, a practice match of the Dutch national team, and the Amsterdam Quad Rugby Tournament, 2017; Wheelchair Tennis: Measured during the 2016 Dutch championship and the international ABN-AMRO wheelchair tennis tournament of 2017.	Wheelchair basketball athletes had the highest average speeds (1.57 ± 0.13 m/s), maximal speeds (4.98 ± 0.43 m/s), and rotational speeds (388 ± 71°/s). Wheelchair tennis athletes had average speeds of 1.34 ± 0.13 m/s, maximal speeds of 4.40 ± 0.40 m/s, and rotational speeds of 369 ± 79°/s. Wheelchair rugby athletes had the lowest average speeds (1.13 ± 0.27 m/s), maximal speeds (3.37 ± 0.99 m/s), and rotational speeds (303 ± 43°/s). Wearable technology is crucial for advancing wheelchair sports.
Chua et al. [22]	IMU: mobile device model: A1367, Apple Inc. (4G iPod touch); sampling rate: 60 Hz	Attached to the frame of the wheelchair	IMU data were collected during six different matches of the Victorian Wheelchair Rugby League (Australia). Data were synchronized and verified against captured video footage. Data analysis involved classifying activities based on sensor data.	Linear acceleration signals were processed using Rényi entropy and Hausdorff dimension, combined into a 2D map identifying five distinct activities. Boundary lines between activities on the 2D map varied with athletes’ classification and skill level, verified with video footage. This method has applications in coaching, match analysis, and talent identification.
Fuss [23]	Electric motor used as a generator; and two 4G iPod touch, sampling rate: 1200 Hz; 60 Hz	Attached to the rear wheels (phone) and frame (electric generator)	Speed profiles were recorded during 100 m training races of four athletes.	Speed profiles give immediate feedback on weak points such as cusps and decreasing velocity due to fatigue. Speed profiles plotted against time can identify weak points and problems causing them, which can be verified with video footage.
Coutts [24]	Magnetic reed switches (180 deg resolution); sampling rate: 1000 Hz	Placed on one rear wheel	Pre-match sessions: included four coast-down trials, where athletes were instructed to reach 75% of their maximal speed before entering the coast-down phase; Additionally, four trials involved maximal acceleration. Match: two athletes participated in a 6 min match.	During the wheelchair basketball match, it was estimated that 64% of the time was spent in propulsive action and 36% in braking activity. Projections for a complete 40 min game indicated that subjects would travel about 5 km at an average speed of 2 m/s.

**Table 3:** Study parameters.

Author [Ref’]	Sensor Data	Key Calculated Parameters	Total Number of Reported Parameters
Fuss and Ow [16]	Wheelchair translational velocity (from motor back-EMF)	Velocity Peak push acceleration Push frequency Forces (inertial, drag, and friction) Peak push power Energy	6
Van der Slikke et al. [17]	Wheel rotation; wheel acceleration; frame rotation; frame acceleration	Average of the best five rotational speeds in a turn Average rotational acceleration Average linear acceleration in the first 2 m from a standstill Average linear speed Average rotational speed in a curve (with forward speed greater than 1.5 m/s) Average of five best linear speeds	6
Rietveld et al. [18]	Wheel rotation, wheelchair rotation (wheelchair tennis)	Rotations to racket side in the following: Curves Turns Linear accelerations Rotations to non-racket side in: Turns Curves Linear velocities	6
Mason et al. [19]	Wheel rotation (wheelchair rugby)	Measured over both 1 s (MDL-1) and 5 s (MDL-5) intervals: Distance covered—total distance the wheelchair traveled Mean speed—average speed Peak speed—highest speed achieved	3
Sporner et al. [20]	Wheel rotation (wheelchair basketball and wheelchair rugby)	Distance covered: Total distance covered during match play Average speed: Average speed throughout the match Number of stops/starts during the match Activity time	4
Van der Slikke et al. [21]	Wheel rotation; wheel acceleration; frame rotation; frame acceleration (wheelchair basketball, wheelchair rugby, and wheelchair tennis)	Average linear speed: The overall average speed across all measurement sections. Average best linear speed: The average speed of the best five runs or speed sections. Average linear acceleration: The average acceleration measured over the first 2 m from a standstill. Average rotational speed: The average rotational speed during curves where the forward speed is above the average speed. Average best rotational speed: The average of the best five turns’ rotational speeds where the forward speed is below the average speed. Average rotational acceleration: The overall average rotational acceleration.	6
Chua et al. [22]	Frame acceleration (wheelchair rugby)	No activities Low activities High-speed coasting High-speed pushing Extreme collisions Activity pie charts Performance–match time curves	7
Fuss [23]	Wheel angular velocity (gyroscope, iPod touch); wheelchair translational velocity (motor back-EMF); wheelchair racing	Velocity profiles Wheelchair acceleration profiles Stroke frequency Stroke consistency Push and recovery time Ratio of push to recovery time	6
Coutts [24]	Wheel rotation (wheelchair basketball)	Average speed Peak speed Acceleration Force Power Propulsive time (% of total time) Braking time (% of total time) Average propulsive power Average braking power Positive work in 40 min game Negative work in 40 min game Total distance in 40 min game	12

**Table 4:** Quality assessment questions.

Question	Fuss and Ow [16]	Van der Slikke et al. [17]	Rietveld et al. [18]	Mason et al. [19]	Sporner et al. [20]	Van der Slikke et al. [21]	Chua et al. [22]	Fuss [23]	Coutts [24]
**Q1.** Is the hypothesis/aim/objective of the study clearly described?	Y	Y	Y	Y	Y	Y	Y	Y	Y
**Q2.** Are they clearly described in the Introduction or Methods?	Y	Y	Y	Y	Y	Y	Y	Y	Y
**Q3.** Are the characteristics of the participants clearly described (including age and sex)?	Y	Y	Y	Y	Y	N	N	N	N
**Q4.** Are the sensor characteristics clearly described?	Y	N	Y	Y	N	N	Y	Y	Y
**Q5.** Are the participant selections described and appropriate?	Y	Y	Y	Y	Y	Y	Y	Y	Y
**Q6.** Are the main findings of the study clearly described?	Y	Y	Y	Y	Y	Y	Y	Y	Y
**Q7.** Are estimates of the random variability in the data for the main outcomes provided?	Y	Y	Y	Y	Y	Y	Y	Y	Y
**Q8.** Is/Are the participant/s representative of the entire population from which they were recruited?	Y	Y	Y	Y	Y	Y	Y	Y	Y
**Q9.** Are the setting and conditions typical for the population represented by the participant/s?	Y	Y	Y	Y	Y	Y	Y	Y	Y
**Q10.** Are the statistical tests used to assess the main outcomes appropriate?	Y	Y	Y	Y	Y	Y	Y	Y	Y
**Q11.** Are the main outcome measures used accurate (valid and reliable)?	Y	Y	Y	Y	Y	Y	Y	Y	Y
**Q12**. Have test–retest reliability and minimum detectable change values of the sensors been reported?	N	N	N	N	N	N	N	N	N
**Q13.** Is a sample size justification, power description, or variance and effect estimate provided?	N	N	N	N	N	N	N	N	N

**Note:** Y = Yes; N = No.

## 3. Results

### 3.1. Search Results

**Figure 1:**
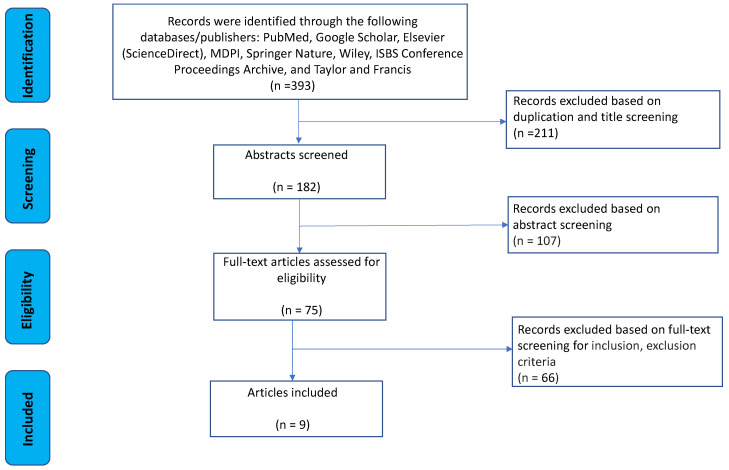
The strategy of the literature review process.

The systematic review identified nine manuscripts [16,17,18,19,20,21,22,23,24] that utilized various motion sensor methodologies to measure different wheelchair performance parameters during training and match time. An initial literature search of databases and publishers yielded a total of 393 potential articles. After removing 211 duplicate references and performing a title screening, 182 citations remained for abstract screening. Of these, 107 citations were rejected for not meeting the inclusion criteria or for meeting the exclusion criteria, leaving 75 citations for full-text eligibility analysis. Finally, a total of nine publications were included in this systematic review (Figure 1).

### 3.2. Study Characteristics

Table 1 summarizes the studies on performance diagnostics in wheelchair sports, highlighting diverse aims, sport types, and participant details. All the studies aimed to obtain a variety of performance parameters for wheelchair sports during match time. One study [16] developed a cost-effective system for monitoring wheelchair velocity during racing, while another created a display of key mobility features for basketball [17]. Research in wheelchair tennis [18] identified key IMU-based mobility components across different player groups. A study in wheelchair rugby [19] compared various tracking systems to measure mobility performance. Additional studies focused on comparing activity profiles in wheelchair basketball and rugby [20], evaluating wearable devices across multiple sports [21] and developing fractal dimension analysis methods for activity identification [22]. Two manuscripts [23,24] examined speed measurement techniques in racing and collected performance data in basketball.

The research covered a variety of wheelchair sports, including wheelchair racing [16,23], basketball [17,20,21,24], tennis [18,21], and rugby [19,20,21,22]. The participants’ experience varied widely, enhancing the generalizability of the findings. For instance, one study included a T53 multiple-medal-winning athlete at international competitions and a T52 athlete with ASEAN Para Games gold medals [16]. Another study involved national and international basketball players [17]). Tennis participants holding ITF rankings [18] were included in one study, while elite male rugby players were featured in another study [19]. The studies on wearable performance tools [20,21] included military veterans and elite athletes across multiple sports (basketball, rugby, and tennis). One study included high-pointer to low-pointer rugby athletes [22], while Paralympic-level racing athletes were examined by another study [23]. National-level basketball players participated in specific trials [24]. The total population sample sizes ranged from 2 to 76, encompassing adult and youth athlete players.

### 3.3. Study Methods and Main Findings

Table 2 provides a detailed summary of the wheelchair sensor type and specifications, location, assessment protocol, and main findings of the studies. The studies employed various sensor technologies, including an electric motor (ACC337), used as a generator at 1200 Hz [16], IMUs from X-IO technologies at 100 Hz or unspecified frequencies [17,18,21], a miniaturized data logger with magnetic reed switches at 8 Hz [19], MDLs with unspecified sampling rates [20], IMUs from X-IO technologies and Shimmer3 [21], an IMU (Apple Inc, model A1367) at 60 Hz [22], smartphones with inbuilt gyroscopes and an electric motor [23], and magnetic reed switches at 1000 Hz [24]. The sensors were attached to various parts of the wheelchair, including the frame [16,22], rear wheels axis and rear frame bar [17,19,21], axle of main wheels [19], wheel spokes [20], or rear wheels only [23,24]. The assessment protocols varied across the studies. Some studies measured kinematics during competitions and friendly matches [17,21] or during specific events like tennis tournaments [18] and simulated rugby matches [19]. Other studies recorded data during training sessions, races, and pre-match activities, such as sprints, training races, and short matches [16,20,22,23,24].

The main findings highlighted key aspects of wheelchair sport performance. Fuss and Ow [16] found that a T53 athlete reached a maximum velocity of 8 m/s, produced six times the peak push power, and generated 2.7 times more energy than a T52 athlete. Van der Slikke et al. and Rietveld et al. [17,18] identified key kinematic outcomes/components that describe wheelchair mobility performance. Another study [19] found no significant differences in mean speed or distance between a MDL and an indoor tracking system, but the MDL underestimated peak speeds. Sporner et al. [20] found significant differences in mobility patterns between basketball and rugby, with basketball showing higher average speeds and distances. Van der Slikke et al. [21] found that basketball athletes had the highest speeds, while rugby athletes had the lowest, highlighting the importance of wearable technology. Chua et al. [22] identified five distinct activities using the translational acceleration signals processed with Rényi entropy and Hausdorff dimension. Fuss [23] provided feedback on weak points and performance issues using speed profiles. Coutts [24] found that 64% of the match time was spent in propulsive action and 36% in braking, with projections for a complete game indicating travel of about 5 km at an average speed of 2 m/s.

### 3.4. Calculated Performance Parameters

Table 3 shows the sensor kinematic data and key calculated parameters reported by all the selected research manuscripts. The studies extracted various sensor kinematic data types from sensors placed on the wheels, focusing on measuring wheel rotation speed [17,18,19,20,21,23,24] (angular velocity) and translational acceleration [17,21]. The sensors were also located on the wheelchair frame bar, calculating the velocity from the back-EMF (electromotive force) generated by an electric motor [16,23], frame rotation [17,18,21] (angular velocity), and frame acceleration [17,21,22,23].

Fuss and Ow [16] calculated six parameters, including translational velocity and acceleration, power, and related wheelchair forces (inertial, drag, and friction). Similarly, three studies, conducted by Van der Slikke et al. [17,21] and Rietveld et al. [18], calculated six parameters, including angular and translational velocities and accelerations in wheelchair tennis, rugby, and basketball sports. Mason et al. [19] and Fuss [23] recorded several key parameters among distance, speed, stroke frequency, and push and recovery time. Sporner et al. [20] measured four parameters, including average speed and start/stop times throughout the match. Chua et al. [22] classified five activity types related to coasting, pushing, and collusion using acceleration data. Coutts [24] measured 12 parameters, including speed, force, power, and distance, over a game’s duration.

### 3.5. Results of the Methodological Quality Assessment

Table 4 summarizes the quality assessment of the studies on performance diagnostics in wheelchair sports, evaluating aspects of study design and reporting. All studies [16,17,18,19,20,21,22,23,24] clearly described their hypotheses, aims, or objectives, as well as their Introduction or Methods (Q1, Q2). Participant characteristics, including age and sex, were generally well described, except in four studies [21,22,23,24], which lacked mean age and gender information. The sensor characteristics were clearly described in most of the studies, though the studies by Van der Slikke et al. [17], Sporner et al. [20], Van der Slikke et al. [21], and Fuss [23] did not specify data collection sampling rate frequencies (Q4). Participant selection methods were appropriate and well described across all studies (Q5). The main findings were presented in all studies (Q6), and estimates of random variability in the data for the main outcomes were provided (Q7). The participants were representative of the populations from which they were recruited, and the settings and conditions were typical for these populations (Q8, Q9). The statistical tests used to assess the main outcomes were deemed appropriate in all studies (Q10), and the main outcome measures used were considered accurate, valid, and reliable (Q11). However, none of the studies that used an IMU device reported test–retest reliability or minimum detectable change values in the sensors (Q12), or provided a sample size justification, power description, or variance and effect estimates (Q13). This assessment highlights the strengths and areas for improvement in the design and reporting of performance diagnostics studies in wheelchair sports, emphasizing the need for comprehensive sensor data reporting and justification of sample sizes to enhance study robustness.

## 4. Discussion

Real-time data collected during wheelchair sport training and competitions provide valuable insights into the game’s practical dynamics and athletes’ performance parameters [15]. While multiple wheelchair studies have investigated controlled-environment protocols during standardized training regimes using treadmills and force plates, studies on real competition and training analyses are relatively limited. This systematic review reports an updated framework of the current research on the use of sensor technology for performance analysis in wheelchair sports during match and training time. This review shows how differently wheelchair sports have used sensor technologies, placed on the wheelchair, to calculate a variety of performance parameters in actual competition and training environments.

Our results show that the use of IMUs, electric motors/generators, magnetic reed switches, and MDLs is promising for quantifying athletes’ wheelchair characteristics during wheelchair sport competitions and training. The results identified nine studies [16,17,18,19,20,21,22,23,24] published between 1992 and 2024. This review covered a variety of wheelchair sports, including racing [16,23], basketball [17,20,21,24], tennis [18,21] and rugby [19,20,21,22]. Participants’ characteristics, such as age and experience level, varied from talented youth tennis players [18] to international gold medal-level professional athletes [16], indicating the generalizability of the findings. Although the studies used different sensor technologies, IMUs were the most common device for collecting kinematic data, including stand-alone IMUs or built-into-smartphone sensors. For example, Rietveld et al. [18] collected wheels and wheelchair rotation data in wheelchair tennis using three IMU sensors located on the hub of each wheel and the frame bar of the wheelchair. The study outcome described six key outcome variables that effectively show the movement capabilities of wheelchair tennis players during a match.

Table 5 shows both the benefits and challenges of different wheelchair sensor technologies for monitoring performance. Many sensors are lightweight, easy to install, and can capture motion data in real-time, such as angular velocity and translational acceleration. However, common limitations include the need for accurate data time synchronization when using multiple devices and less accuracy at higher speeds or with low-resolution sensors. Calibration and time needed for processing the data are also frequent issues that affect how reliable and efficient the data collection is.

The results also show that sensors were placed at different locations, including on one or two wheels and the frame bar of the wheelchair, to record several kinematic sensor data, such as wheel and wheelchair rotation (angular velocity), wheel (angular) acceleration, and wheelchair translational velocity and acceleration. For example, Van der Slikke et al. [21] placed three sensors on two rear wheel axes and on the rear frame bar to evaluate the effectiveness of IMUs for monitoring the wheelchair mobility performance of wheelchair tennis, rugby, and basketball athletes during match play.

**Figure 2:**
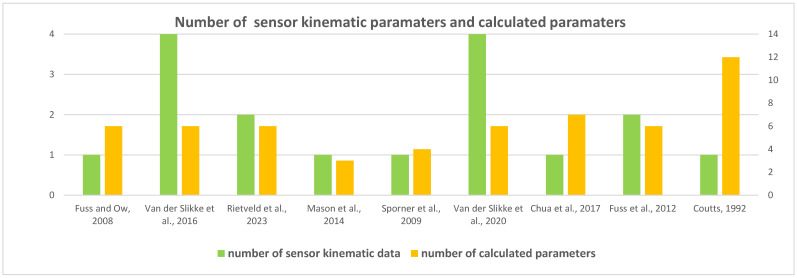
Summary of sensor kinematic data and calculated parameter number from selected studies [16,17,18,19,20,21,22,23,24].

From these sensor kinematic data, the studies calculated performance parameters such as match-time translational and/or angular wheelchair velocity summary data and other performance parameters, for example, the total distance, as well as wheelchair propulsion acceleration, force, power, and number of stops/starts, during the period of the match. Figure 2 provides a summary of the number of sensor kinematic data, e.g., wheel rotation (angular velocity) or wheelchair acceleration, for their respective calculated parameters and the total number of parameters per study, highlighting that studies predominantly focused between one and four sensor kinematic data types to calculate 3–12 key parameters for their respective analysis. For example, Coutts [24] calculated 12 parameters, including speed, force, power, and distance, collected from a wheel magnetic reed switch (180 deg resolution; 1000 Hz) in wheelchair basketball players during game conditions. Their study concluded that during the wheelchair basketball match, it was estimated that 64% of the time was spent in propulsive action and 36% in braking activity and projections for a complete 40 min game, which indicated that subjects would travel about 5 km at an average velocity of 2 m/s.

**Table 5:** Summary description of sensor system technologies used to monitor performance.

Author	Sensor Type	Sampling Rate Frequency	Sensor Placement	Sport	Sensor Physical Quantity	Sensor Units	Sensor Error	Advantages	Limitations
Fuss and Ow [16]	Electric motor used as a generator	1200 Hz	Frame of the wheelchair, connected to the rear wheel by a small wheel	Racing (100 m)	Motor back-EMF to angular velocity to translational velocity	Voltage generated by the motor converted to velocity in m/s	1% unfiltered signal; approx. 0.1% filtered signal	Measures velocity with high precision; simple calibration	Single sensor; not suitable for turns; installation requires a small additional wheel in contact with the rear wheel
Van der Slikke et al. [17]	IMU	Not specified	Rear wheels axis, rear frame bar	Basketball	Angular velocity, translational acceleration	deg/s, m/s^2^	Skid correction applied to reduce the error	Accurate measurement of angular velocity	Data fusion and time synchronization when using multiple devices was not reported
Rietveld et al. [18]	IMU	100 Hz	Hub of each wheel, frame bar	Tennis	Angular velocity, translational acceleration	deg/s, m/s^2^	Not reported	Accurate measurement of angular velocity	Data fusion and time synchronization when using multiple devices was not reported
Mason et al. [19]	Miniaturized data logger and magnetic reed switches	10 Hz	Magnet attached near the axle of the wheels	Rugby	Angular velocity	Interval counts (120 deg resolution)	Not reported	High sensitivity to magnetic fields	Low resolution (120 degrees); lower accuracy at higher speeds
Sporner et al. [20]	Miniaturized data logger and magnetic reed switches	Not reported	Magnet attached to wheel spokes	Basketball, Rugby	Angular velocity	Interval counts (120 deg resolution)	Not reported	High sensitivity to magnetic fields	Low resolution (120 degrees); lower accuracy at higher speeds
Van der Slikke et al. [21]	IMU	Not specified	Rear wheel axis and rear frame bar	Basketball, Rugby, Tennis	Angular velocity, translational acceleration	deg/s, m/s^2^	Skid correction applied to reduce the error	Accurate measurement of angular velocity	Data fusion and time synchronization when using multiple devices was not reported
Chua et al. [22]	iPod Touch IMU	60 Hz	Frame of the wheelchair	Rugby	Acceleration	m/s^2^	Not reported	Lightweight, small, and easy to install	Acceleration range restricted to ±2.25 *g*
Fuss [23]	Electric motor used as a generator; iPod Touch IMU	1.2 kHz; 60 Hz	Frame of the wheelchair rear wheels	Racing (100 m)	Motor back-EMF to angular velocity to translational velocity; angular velocity	Voltage generated by motor converted to velocity in m/s; deg/s	1% unfiltered signal; approx. 0.1% filtered signal	Measures velocity with high precision; simple calibration (both sensor systems)	Single sensor; not suitable for turns; electric motor installation requires a small additional wheel in contact with the rear wheel
Coutts [24]	Magnetic reed switches	1000 Hz	Rear wheel	Basketball	Angular velocity	Intervals (180 deg resolution)	Not reported	High sensitivity to magnetic fields	Low degree resolution (180 degrees); lower accuracy at higher speeds

Some of the limitations in this review are related to significant differences in sport types, athletes’ levels and ages, and match and training duration. Also, there was poor reporting on test–retest reliability and minimum detectable change values in studies that used IMU devices, and none of the studies provided sample size justification, power description, or variance and effect estimates.

## 5. Future Directions

The shift from pure research to the general practical application of sensors in wheelchair sports is long overdue. The focus of this systematic review is to provide the current state of the art of practical sensor applications in training and match settings. While pure research in this area aims to explore data related to staged drills (e.g., [5,28,29,30,31]), the practical application of sensors in training sessions and competitions provides processed data to coaches and high-performance managers for decision-making. The latter serves for comparing athletes and their day-to-day performance fluctuations, player selections for national teams, and player assessment and classification [22]. Despite the demand for practical applications of sensors, the inherent research is unlimited, and focuses, e.g., on the discovery of new performance parameters, improvement in signal analysis methods (incl. artificial intelligence), match data analytics, big data management, visualization of performance parameters and statistics, and effective condensing of the most essential information for the benefit of the athletes and coaches. However, to make the best use of sensor technology in such applications within wheelchair sports, several practical challenges still need to be addressed. Future research should focus on the following areas:

- To ensure accurate data when using sensors like IMUs on both wheelchair wheels or multiple wheelchairs, precise time synchronization is critical, especially since the sensors are not physically connected (e.g., using SD cards or wireless data transmission). Millisecond-level accuracy in syncing timestamps across all sensors and tracking systems is needed to keep the recorded data reliable and correct.
- Improving IMUs to handle the physical demands, such as high impacts and collisions, of wheelchairs ensures they are durable and reliable during intense match activity. Furthermore, making these devices easy to set up on wheelchairs and to use in both training and competitions will improve their practicality for coaches and athletes. Furthermore, misalignments of sensors, especially IMUs, should be avoided or at least corrected by calibration [32]. These misalignments can occur when the IMUs are mounted on the spokes, which are oriented at an angle to the plane of the wheel.
- Future research should focus on developing standardized protocols for using sensors in wheelchair sports. These standardized protocols research should continue the work of Camomilla et al. [33], who reviewed the use of wearable sensors in sport performance evaluation and highlighted their potential when supported by standardized protocols. Rupf [34] demonstrated that a single IMU can reliably measure wheelchair kinematics in elite athletes, suggesting that standardization could enhance data accuracy. In addition, Li et al. [35] reinforced the need for consistent guidelines to improve athletic performance and prevent injuries. By adopting these protocols, wearable sensors in wheelchair sports can be used more effectively for performance evaluation and injury prevention.
- Three-dimensional visualization and augmented reality (AR) technologies, combined with IMU data, can improve how athletes and coaches work with performance, movement, and game strategies. IMUs can give real-time motion insights, and with AR, athletes receive instant visual feedback to help them adjust during practice and training. Coaches can also provide real-time, data-based feedback to improve specific aspects of performance. These tools are useful for both training and reviewing after competitions, making data easier to manage and helping athletes and teams make better decisions.

## 6. Conclusions

This systematic review highlights the successful integration of various sensor technologies in wheelchair sports to calculate athlete performance parameters during match time. Despite the diversity in study objectives and methods, most studies demonstrated that sensor-derived data effectively met their goals. However, inconsistencies in reporting reliability and sample size justification indicate a need for standardized protocols. Future developments and research in this field should focus on the general application of sensors in wheelchair sports as a gold standard for providing real-time match data and deeper insights into performance metrics. Overall, advancements in sensor technology offer an impactful potential for enhancing wheelchair sport performance through detailed performance diagnostics to improve individual and whole-team strategies and performance.

## Data Availability

All data generated in this review can be found in the text.
